# Research Progress on Template Methods for Perovskite Nanomaterials in Solar Energy Conversion

**DOI:** 10.3390/molecules31152586

**Published:** 2026-07-24

**Authors:** Jinran Li, Zhenrui Du, Jie Du

**Affiliations:** College of Materials Science and Engineering, Hainan University, Haikou 570228, China; 20243008675@hainanu.edu.cn (J.L.); 20223000742@hainanu.edu.cn (Z.D.)

**Keywords:** perovskite nanomaterials, template method, solar energy conversion, crystal growth, photovoltaic materials

## Abstract

Perovskite nanomaterials possess superior optoelectronic characteristics, flexible structural tunability, simple synthesis routes and low manufacturing costs, making them outstanding candidates for solar energy conversion. Nevertheless, their practical commercialization is greatly restricted by intrinsic drawbacks such as poor environmental stability, difficulties in large-area fabrication and potential environmental risks. As an efficient and feasible strategy to overcome these bottlenecks, the template method utilizes the spatial confinement and interfacial induction effects of templates to precisely modulate the nucleation, growth, morphology and crystalline quality of perovskite nanomaterials. This review systematically summarizes the recent advances in template-regulated synthesis of perovskite nanomaterials for solar energy applications. It comprehensively elaborates the regulatory mechanisms and representative research progress of inorganic, organic and composite templates in perovskite preparation, focusing on their unique effects on optimizing the crystalline quality and operational stability of perovskite photovoltaic materials. The current technical limitations and future development directions of template method are also discussed. This review aims to provide a reliable theoretical basis and practical guidance for the rational design, controllable fabrication and performance optimization of high-efficiency and stable perovskite materials for solar energy conversion.

## 1. Introduction

Perovskite materials are a class of functional materials with an ABX_3_ crystal structure. Their exceptional optoelectronic properties, strong structural tunability, relatively straightforward preparation processes and low production costs collectively endow them with immense potential in various fields, particularly in solar energy conversion, as well as in electronic information technology, photodetection and light-emitting devices. Among these applications, perovskite nanomaterials have emerged as core components for high-performance perovskite solar cells (PSCs), a cutting-edge third-generation photovoltaic technology that has achieved remarkable progress since its initial report in 2009—single-junction laboratory efficiencies have soared from 3.8% to over 27%, and perovskite–silicon tandem cells have reached 34.85%, surpassing the theoretical limit of single-junction silicon solar cells [1,2,3,4]. 

To further advance perovskite nanomaterials and promote their commercial application in solar energy conversion, the crystalline quality of these materials is paramount. The state of perovskite crystals directly determines carrier transport efficiency and environmental stability, which are the two core performance indicators of PSCs. Consequently, developing low-cost, high-efficiency methods for producing high-quality perovskite nanocrystals has become the key to driving their widespread adoption in solar energy conversion [5,6,7,8,9,10]. Despite the great potential of perovskite nanomaterials in photovoltaic applications, they still face critical challenges, including poor structural order, high defect density, and inadequate environmental stability, which lead to rapid performance degradation of PSCs under harsh conditions (e.g., high temperature, high humidity, and UV irradiation) and hinder their large-scale commercialization. In this context, the template method has emerged as a highly promising strategy to address these bottlenecks. 

To achieve the goal of low-cost, high-quality perovskite nanocrystal synthesis for solar energy conversion, the template method leverages the spatial confinement effect and interfacial interaction of templates to precisely regulate the nucleation and growth processes of perovskite nanomaterials, thereby enabling accurate control over crystal morphology, size, orientation, and defect density [11,12,13,14,15,16]. This review provides a detailed overview of the development and application of the template method in perovskite nanomaterial synthesis for solar energy conversion. It first elaborates on the fundamental principles of the template method, including how templates provide heterogeneous nucleation sites to inhibit random crystal growth and guide oriented growth. Then, it discusses the latest research progress in the template method, focusing on how this strategy optimizes the optoelectronic properties of perovskite nanomaterials, enhances carrier transport efficiency, and improves the long-term stability of PSCs. Finally, it outlines the future development directions of the template method, such as optimizing template design, developing scalable template-based fabrication processes, and integrating templated perovskite nanomaterials into tandem solar cell architectures, which are crucial for accelerating the commercialization of PSC technology and supporting the global transition to renewable energy and carbon neutrality goals. 

## 2. Overview of Perovskite Materials

**Figure 1:**
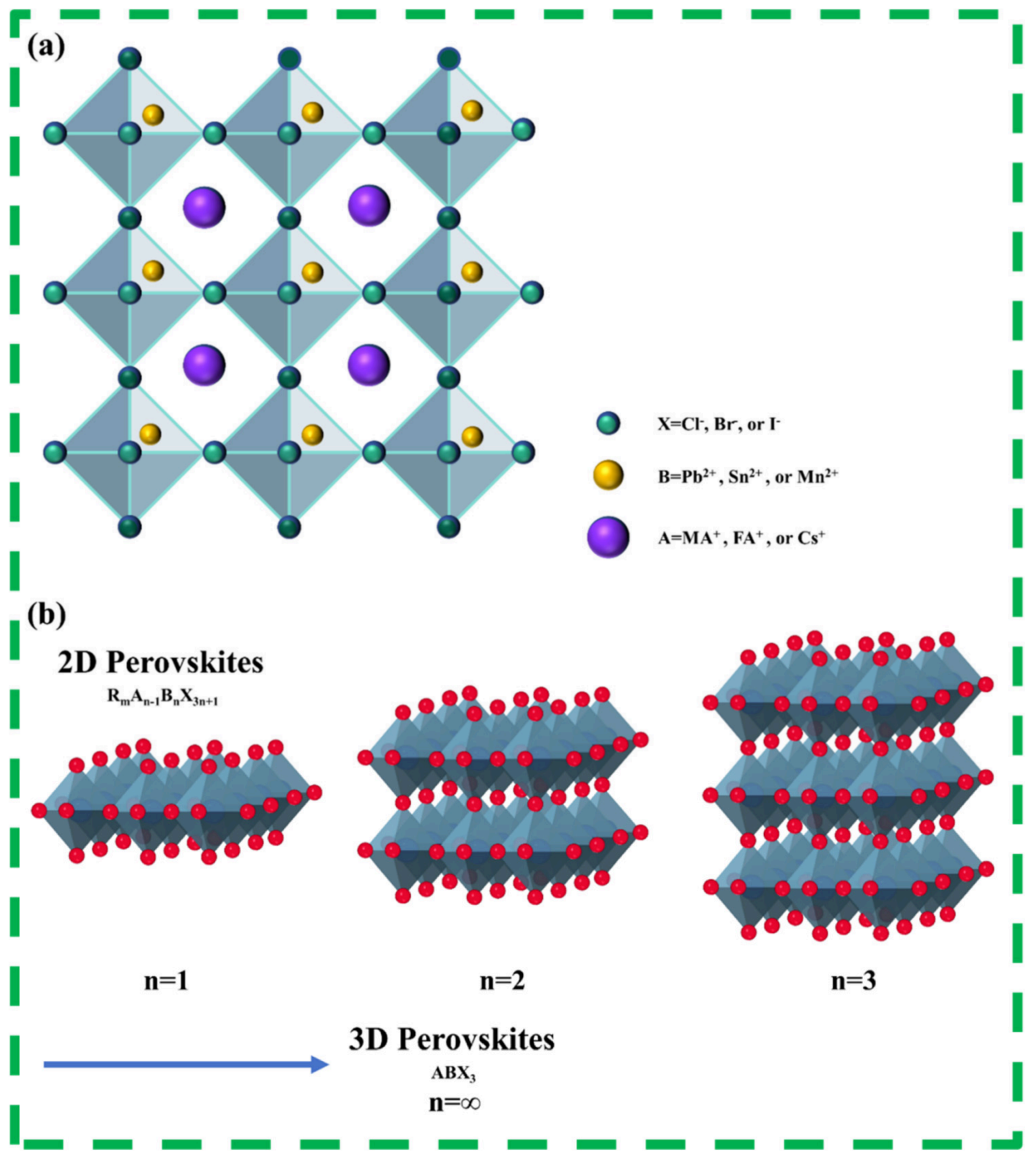
Crystal structure diagrams of perovskite materials. (**a**) Schematic representation of the typical three-dimensional perovskite structure ABX_3_. Cation A is incorporated into the body-centered position of a cubic framework composed of eight co-vertex [BX_6_]^4−^ octahedra. (**b**) Schematic diagrams of perovskite materials with varying dimensionalities.

Perovskite materials possess a typical ABX_3_ crystal structure, in which A-site cations include organic FA^+^, MA^+^ and inorganic Cs^+^; B-site mainly consists of divalent Pb^2+^ and Sn^2+^; and X-site corresponds to halide anions such as Cl^−^, Br^−^ and I^−^. The basic framework is constructed by vertex-sharing BX_6_ octahedra, and lattice distortion enables diversified low-symmetry configurations. Perovskite systems cover inorganic, organic–inorganic hybrid, double perovskite and low-dimensional subtypes, exhibiting high compositional flexibility and outstanding optoelectronic superiority for solar energy conversion [17,18,19,20,21,22,23]. The schematic crystal structures of 3D and 2D perovskites with different dimensionalities are shown in Figure 1a and Figure 1b, respectively.

**Figure 2:**
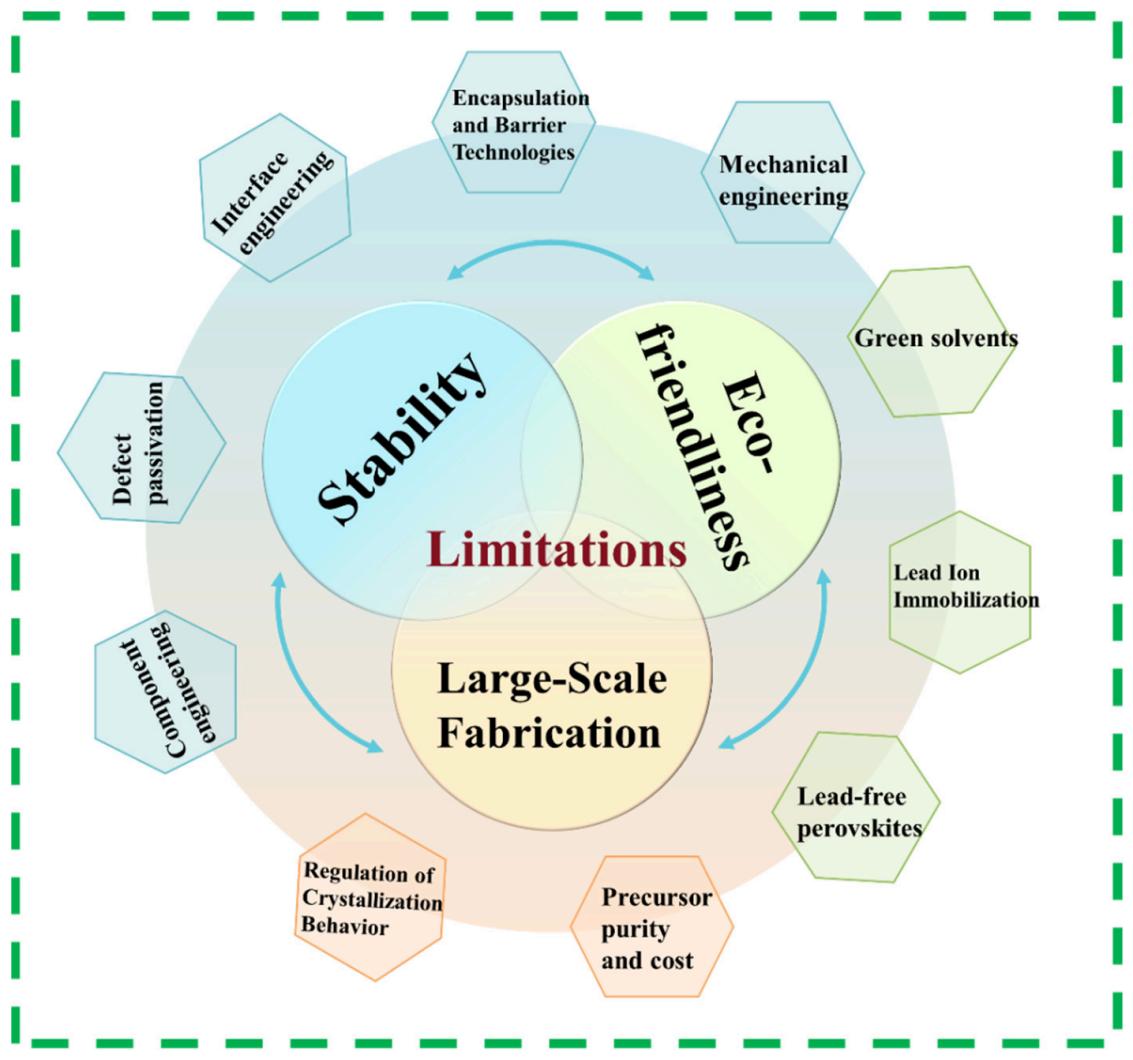
Diagram illustrating key constraints and improvement strategies in the development of perovskite materials.

Despite the remarkable advances in photovoltaic efficiency, perovskite materials still face inherent bottlenecks restricting practical commercialization. Poor environmental and thermal stability, high defect density, unavoidable ion migration and difficulty in large-area uniform fabrication severely degrade the long-term output reliability of devices [14,24,25]. In addition, lead toxicity and insufficient preparation reproducibility further hinder industrial-scale deployment, as summarized in Figure 2.

In this context, the template method has emerged as a facile and effective strategy to address the above challenges. By virtue of spatial confinement, lattice matching and interfacial molecular interactions, templates can precisely regulate perovskite nucleation, oriented growth, grain morphology and defect passivation, providing a feasible route to simultaneously improve crystalline quality, optoelectronic performance and operational stability.

From the perspective of future industrialization, the scalability and cost-effectiveness of template strategies are critical prerequisites for commercial application. Most currently reported high-performance templates rely on expensive raw materials, complex pretreatment, non-recyclable components and rigorous laboratory preparation conditions, which are difficult to transfer to large-area manufacturing. Moreover, many template systems suffer from poor batch reproducibility, high material consumption and incompatibility with mainstream low-cost fabrication techniques such as blade coating and roll-to-roll processing. Clarifying the cost composition, recyclability potential and scalable process compatibility of inorganic, organic and composite templates is therefore essential to promote the real industrial landing of template-modified perovskite photovoltaic technology [26,27,28].

Despite the current intensive research focus on perovskite materials and their considerable potential in photovoltaic and luminescent fields, their inherent shortcomings in environmental stability, large-scale production feasibility and environmental friendliness remain prominent and undeniable [29,30,31,32]. These critical bottlenecks not only impede their practical application and industrialization advancement but also highlight the urgency of continuously refining and innovating improvement strategies to address these challenges and fully exploit their application potential, as shown in Figure 2. 

## 3. Results and Discussion

### 3.1. Introduction to the Template Method

**Figure 3:**
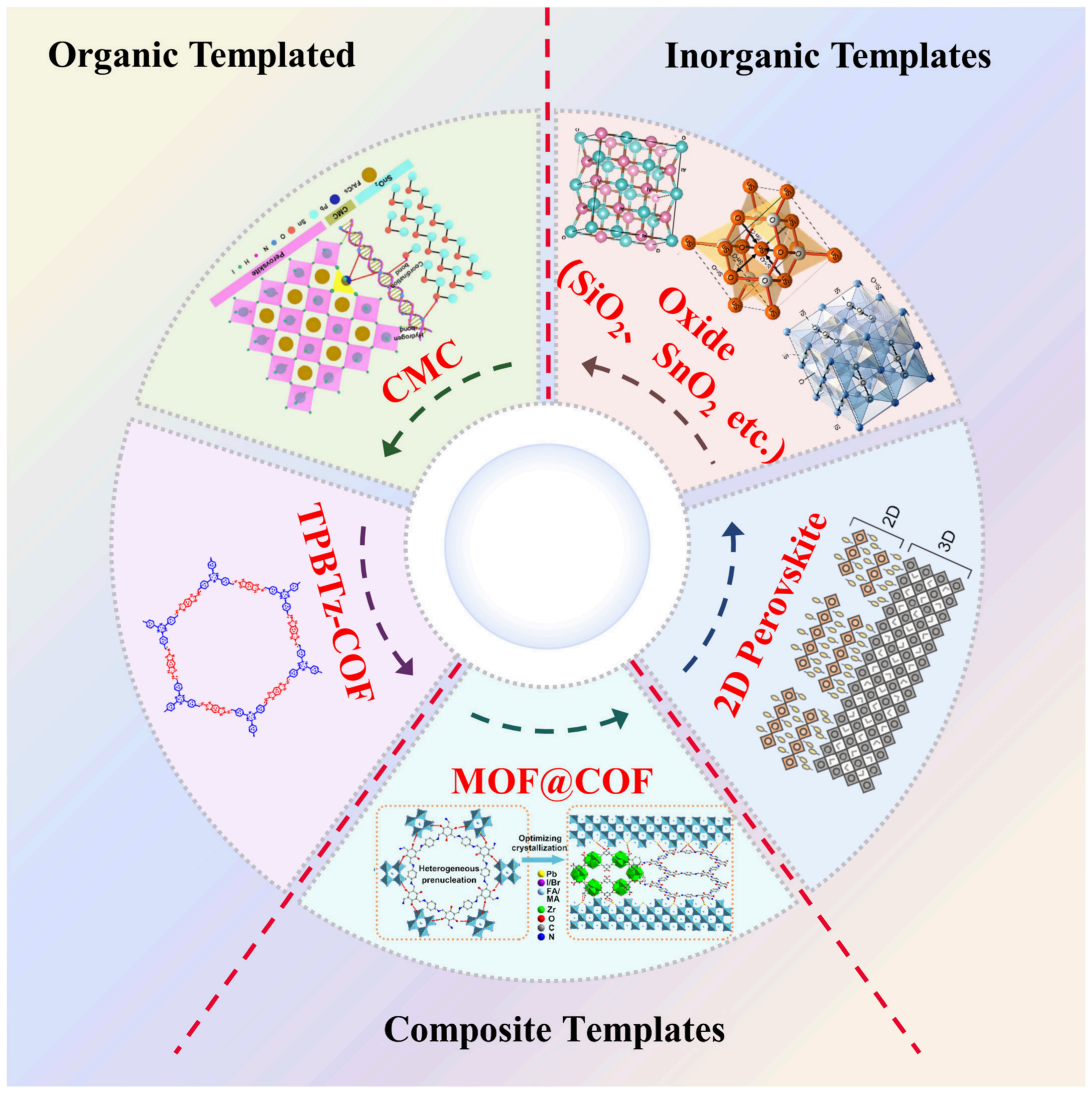
The following schematic diagram illustrates the template method for perovskite materials. In accordance with the principles of composition, it is possible to categorize templates in three primary ways: inorganic, organic and composite.

The templating approach constitutes a preparation and modification strategy that achieves precise control over the size, morphology, crystalline phase, orientation and micro/nanostructure of perovskite precursors. This is accomplished by designing templates with specific structural or chemical properties to confine growth space and provide heterogeneous nucleation sites, thereby guiding nucleation, crystallization and growth [33]. The strategy is centered on the exploitation of the spatial confinement, interfacial induction or molecular recognition effects of templates to achieve the controlled construction of perovskite material structures and properties. The objective is to prepare high-quality, high-performance and functionally specialized perovskite materials. In the following section, the templates will be categorized based on their composition. This will result in three distinct types: inorganic templates, organic templates and composite templates, as shown in Figure 3. The subsequent discussion will elaborate on the achievements attained by researchers worldwide through the template method.

### 3.2. Mechanistic Understanding of Template-Mediated Perovskite Crystallization

In the template-free system, perovskite precursors undergo spontaneous and random nucleation without spatial restriction and interfacial guidance. The resultant polycrystalline films usually suffer from disordered crystal orientation, irregular grain size, high defect density, severe lattice strain, and uncontrollable ion migration, which inevitably degrade carrier transport capability and long-term device stability.

In contrast, inorganic templates rely on spatial confinement, lattice epitaxial matching, and heterogeneous nucleation sites to modulate the entire crystallization process. The rigid framework of inorganic templates constrains the growth space of perovskite grains, induces preferentially oriented crystallization, suppresses the formation of random grain boundaries, and relieves residual lattice strain. Meanwhile, suitable lattice matching between inorganic templates and perovskite lattices reduces interfacial defects, optimizes grain connectivity, and prolongs charge carrier lifetime.

Organic and polymer templates regulate perovskite nucleation and crystal growth mainly through intermolecular non-covalent interactions, including hydrogen bonding, electrostatic attraction, van der Waals forces, and Lewis acid–base coordination. Functional groups on organic molecular chains can anchor perovskite ions, homogenize precursor distribution, and guide uniform nucleation. Such template effects effectively refine grain morphology, passivate undercoordinated Pb^2+^ and halide vacancies, inhibit ion migration, and improve film compactness and mechanical robustness.

Composite templates integrate the merits of inorganic rigid confinement and organic interfacial functionalization. The synergistic effect enables simultaneous regulation of crystal orientation, microstructure evolution, interfacial energy-level alignment, and defect passivation, achieving simultaneous optimization of crystalline quality, optoelectronic performance, and operational stability of perovskite photovoltaic materials. A schematic illustration of perovskite crystallization regulation mechanisms under template-free, inorganic, organic and composite template conditions is shown in Figure 3.

### 3.3. Inorganic Template-Guided Perovskite Materials

Inorganic templates utilize inorganic materials as substrates, leveraging their rigidity and lattice-matching properties alongside specific morphologies to confine perovskite precursor growth, regulate crystal orientation, and enhance nucleation sites. This enables precise structural control of perovskite materials. The inherent high structural stability of inorganic templates is leveraged to offer superior mechanical support, high-temperature resistance, and chemical corrosion resistance. Furthermore, they are characterized by their ease of removal and recyclability, a property that significantly enhances their application value in the field of perovskites.

### 3.4. Oxide-Based Inorganic Templates

Oxides, including SiO_2_ and SnO_2_, are regarded as exemplary raw materials for the purpose of synthesizing inorganic templates in the context of perovskite materials. It has been demonstrated that the aforementioned processes facilitate precise morphology control whilst concomitantly enhancing material stability. When employed in conjunction with established synthesis techniques, these templates have the potential to further extend the scope of perovskite applications because of their cost-effectiveness. For instance, Zhao et al. employed SiO_2_ as both a raw material and inorganic template for perovskite synthesis. The research team was inspired by the efficient circularly polarized light modulation exhibited by biological chiral photonic crystals. To this end, they utilized a chiral nematic phase structure self-assembled from cellulose nanocrystals (CNCs) as the initial template. A mesoporous SiO_2_ rigid template was fabricated through the process of selective removal of CNCs during the SiO_2_ coating procedure. Subsequently, cesium-lead halide perovskite precursors were loaded into the mesopores, thereby enabling the perovskite to replicate the chiral photonic crystal structure of the template. The emission frequency of the perovskite was precisely coupled with the cavity resonance frequency of the photonic crystal within the visible spectrum, thereby achieving circularly polarized luminescence. The fabrication of three-color chiral perovskite photonic crystal composites was successfully achieved, namely blue (CsPbClBr_2_), green (CsPbBr_3_), and red (CsPb_0.83_Zn_0.17_I_3_). Within the template, the perovskite forms a continuous semiconductor phase, thereby satisfying the charge transport requirements for electronic devices. The circularly polarized emission exhibits an asymmetry factor of up to −0.34, with narrow half-widths of 25–57 nm and a maximum quantum yield of 10.23%. This research approach demonstrates that specific circularly polarized light output at targeted wavelengths can be achieved by adjusting both the template’s photonic bandgap and the perovskite composition. This provides a novel pathway for the development of high-polarization-degree chiral luminescent materials and ordered microstructured semiconductors [34].

Anodized aluminum oxide (AAO) has also been demonstrated to serve as an excellent template material, as evidenced by J. Ashley et al. Precisely controlled nanowire diameters (50–200 nm) have been achieved using AAO templates with tunable pore sizes. The resolution of issues pertaining to poor nanowire-pore-bottom contact and surface impurity retention, which are inherent to conventional synthesis methods, was achieved by leveraging the synergistic effects of solvent washing, centrifugation and capillary forces. This approach resulted in nanowires of uniform diameter (with a coefficient of variation of less than 10%) and oriented arrays reaching 80 cm^2^ in area. The method demonstrates excellent compositional versatility, successfully synthesizing both organometallic halides (CH_3_NH_3_PbI_3_, CH_3_NH_3_PbBr_3_) and fully inorganic lead-free perovskite nanowires (Cs_2_SnI_6_, synthesized via templating for the first time). Structural performance studies indicate that nanowires with a diameter of 110 nm exhibit minimal lattice strain and the longest characteristic charge carrier lifetime (~60 ns). This lays a crucial foundation for structural optimization and performance enhancement in perovskite-based optoelectronic devices [35]. 

**Figure 4:**
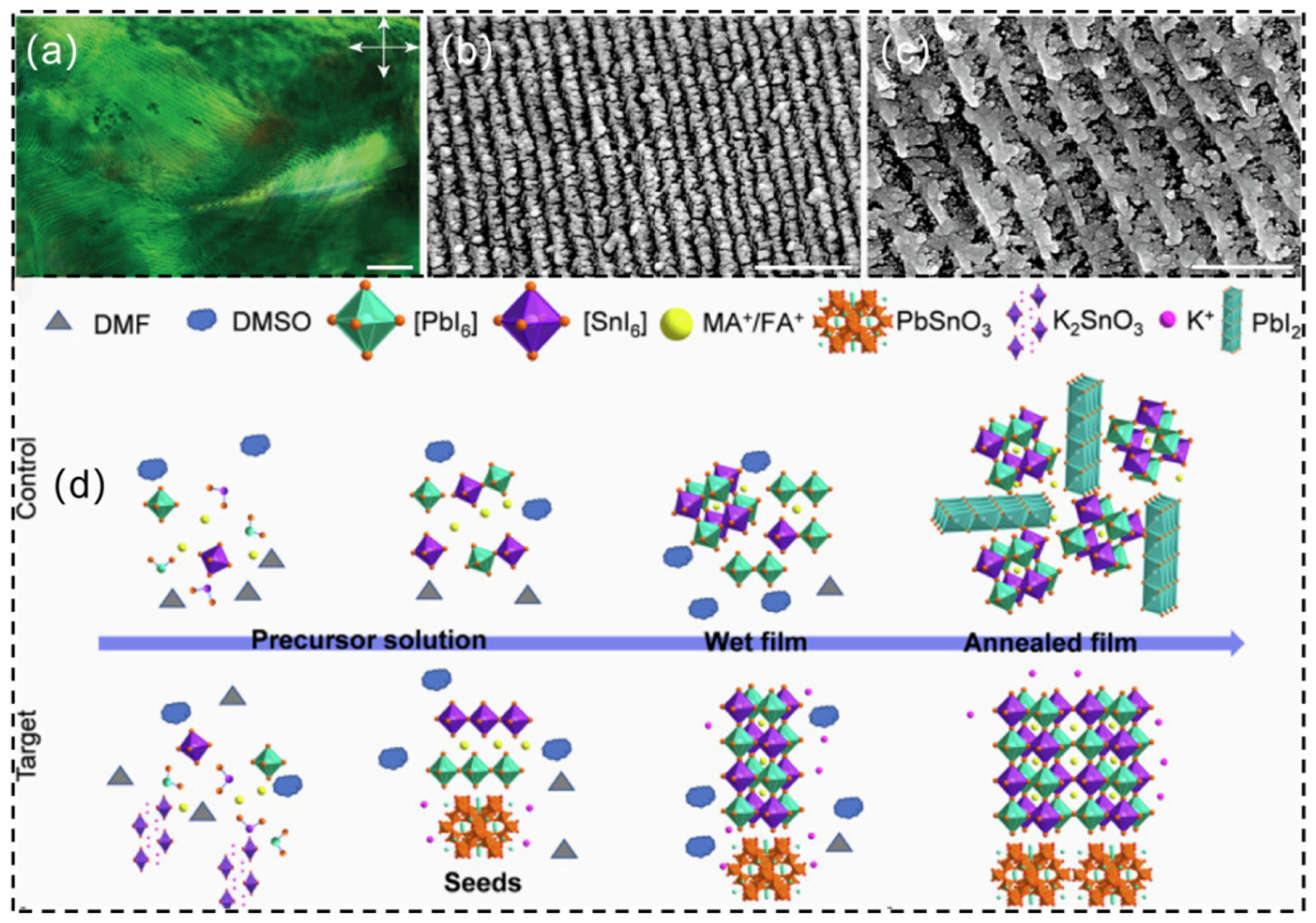
(**a**) POM image showing the fingerprint textures in a PVK_G/Silica_G composite film. (**b**,**c**) Cross-sectional SEM images revealing chiral nematic microstructures inside a PVK_G/Silica_G composite film. Scale bars: 50 µm (**a**), 1 µm (**b**) and 500 nm (**c**) [35]. (**d**) Schematic diagram illustrating the seed-induced oriented crystallization of K_2_SnO_3_-treated FA_0.7_MA_0.3_Pb_0.5_Sn_0.5_I_3_ perovskite films [37].

Further innovative inorganic templates have been extensively applied in perovskite material synthesis [36,37]. For instance, Chen et al. pioneered the template method for regulating halide perovskite growth, operating through the introduction of potassium stannate (K_2_SnO_3_) into perovskite precursors and buried hole transport layers. This induces spontaneous in-situ reactions with lead iodide (PbI_2_), generating ABX_3_^−^ structured PbSnO_3_ nucleation seeds and potassium iodide (KI), where PbSnO_3_ serves as a template, as shown in Figure 4. By leveraging its 98% lattice matching with Sn-Pb perovskite, it guides preferential oriented crystallization of the perovskite, eliminates intermediate phases, and alleviates interfacial stress. Similarly, KI and SnO_3_^2−^ synergistically passivate defects, suppress Sn^2+^ oxidation, and inhibit ion migration. Employing this templating strategy, a single-junction Sn-Pb perovskite solar cell with a 1.25 eV bandgap achieved a steady-state efficiency of 23.12%, while significantly enhancing the stability of the perovskite material. Two-terminal (2T) and four-terminal (4T) all-perovskite tandem cells achieved steady-state efficiencies of 28.12% and 28.81%, respectively. This templating approach proves equally effective for both 1.77 eV wide-bandgap and 1.54 eV conventional bandgap perovskite materials, enhancing their photovoltaic performance and demonstrating broad applicability.

#### Inorganic Templates Based on Two-Dimensional Perovskite Materials

**Figure 5:**
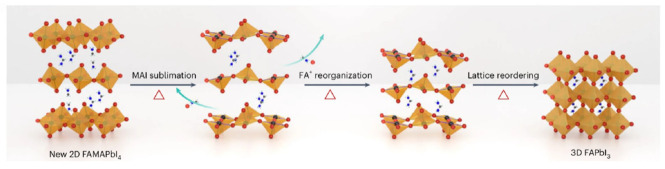
Schematic of the 2D FAMAPbI_4_ to 3D FAPbI_3_ phase transformation. The red triangles under the arrows represent heating [39] Copyright 2025, Springer Nature.

The layered crystal structure and physicochemical stability of two-dimensional perovskite materials have been shown to effectively regulate the nucleation and growth of three-dimensional perovskites. This process has been demonstrated to suppress defect formation and enhance the long-term reliability of devices. For instance, Gao et al. employed the in situ generation of 2D perovskite-assisted controlled growth (2D-ACG) technique. A two-step deposition method was employed to locally embed a top inner layer of 2D perovskite (using (ThPy)_2_ PbI_4_ constructed from the novel organic salt ThPyI as a template) within the film prior to the formation of formamidyl (FA)-based perovskite. The in-plane Pb-I-Pb bond angle (174°) of the resultant material matches α-FAPbI_3_, thereby regulating crystal nucleation and oriented growth, retarding renucleation kinetics, releasing residual strain, and passivating bulk and surface defects. The resulting 2D/3D composite perovskite film exhibits large grain size (exceeding 1 μm), low defect density, and a carrier lifetime exceeding 27 μs. The perovskite solar cell under consideration has been shown to achieve a high efficiency of 26.16% (certified at 25.84%) and a high open-circuit voltage of 1.213 V. It has also been demonstrated to maintain structural stability when subjected to extreme conditions of 200 °C and 75 ± 5% relative humidity. Furthermore, the study demonstrated that unenhanced devices maintained 93% of their initial efficiency after 1350 h at 50 °C and retained 92% of their performance after 100 thermal cycles from −20 °C to 45 °C, thereby achieving synergistic breakthroughs in both efficiency and stability [37]. Concurrently, Liang et al. pioneered an innovative application of two-dimensional perovskites by proposing a “2D-to-3D (α)” all-solid-state phase transition strategy, as shown in Figure 5. Utilizing the 2D layered perovskite (FAMAPbI_4_) as a template, the sublimation of methylammonium iodide (MAI) molecules was induced through mild annealing at 120 °C. This process facilitated the rearrangement of the FA^+^ cation and directed the alignment of the inorganic framework. This process has been shown to result in a solvent-free transformation into highly oriented α-FAPbI_3_ perovskite, which exhibits the lattice orientation characteristics of the 2D template, while concurrently effecting a reduction in lattice mismatch and defect states. The T-FAPbI_3_ films that are the result of this process exhibit single (100) out-of-plane orientation, larger grain size (831 nm), lower trap density, and extended carrier lifetime (581.48 ns). Solar cells fabricated from this material achieve a photoconversion efficiency of 25.01%. Furthermore, unencapsulated devices maintained 90.6% of their initial efficiency after 1200 h of storage in ambient air. Furthermore, large-area modules exceeding 70 cm^2^ with efficiencies surpassing 20% were successfully fabricated, offering a novel pathway towards enhancing the efficiency, stability, and large-scale production of perovskite photovoltaic devices [38]. 

As further advanced by Sidhik et al., the utilization of two-dimensional perovskite templates was found to stabilize the three-dimensional formamidopropyl lead iodide (FAPbI_3_) perovskite α phase (black phase) through lattice-matched two-dimensional (2D) perovskite templates. Specifically, the employment of butylammonium (BA) or pentylammonium (PA) as bulky cations in Ruddlesden–Popper phase 2D perovskites (BA_2_FAPb_2_I_7_, PA_2_FAPb_2_I_7_) was found to be particularly effective. It is evident that these exhibit near-perfect lattice matching with the (011) plane spacing of 3D FAPbI_3_, as well as the (001) interplanar spacing of 3D FAPbI_3_. The addition of 0.5–1.0 mol% of the pre-synthesized 2D perovskite powder to the FAPbI_3_ precursor solution is required. The lower formation enthalpy and room-temperature phase stability of the 2D perovskite were leveraged, resulting in preferential nucleation. The use of a lattice template effect resulted in the preferential formation of the α phase of FAPbI_3_ at 100 °C, which is significantly lower than the conventional 150 °C annealing temperature. In turn, it suppressed the transition to the non-photoactive δ phase via mild compressive stress. The resulting FAPbI_3_ perovskite material exhibited remarkable characteristics, including a narrow bandgap of 1.48 eV (approaching the theoretical limit), excellent crystalline quality, and reduced non-radiative recombination. This material demonstrated a high photovoltaic conversion efficiency of 24.1% in a 0.5 cm^2^ active area p-i-n inverted structure device. The study demonstrated unparalleled stability, with unencapsulated devices maintaining 99% efficiency retention after 500 h at 60 °C under AM1.5G illumination and MPPT conditions. Encapsulated devices exhibited a 97% efficiency retention after 1000 h under the stringent ISOS-L-2 conditions of 85 °C and 70% relative humidity. This breakthrough represents a significant departure from the conventional trade-off between “efficiency and stability” [39]. 

Furthermore, the precise regulation of vertical orientation of 2D perovskite templates is critical for optimizing the carrier transport and interface contact of 3D perovskite absorbers. Controlled growth of vertically aligned 2D perovskite layers can establish continuous out-of-plane charge transport channels, effectively suppress grain boundary recombination, and minimize lattice mismatch at the 2D/3D heterointerface. Recent advances have demonstrated that rational molecular design and interface engineering can precisely modulate the stacking mode and crystal orientation of 2D perovskites, enabling highly vertical crystal arrangement and favorable lattice matching with 3D perovskite frameworks [38]. Such vertically oriented 2D templates not only guide the oriented growth of upper 3D perovskite grains but also act as efficient hole/electron transport bridges, simultaneously optimizing interfacial energy-level alignment and long-term device stability.

### 3.5. Organic Template-Assisted Perovskite Materials

Beyond inorganic templates, organic templates represent a highly competitive alternative for perovskite material synthesis. They guide perovskite crystal growth and modulate crystalline phase structure through intermolecular interactions (e.g., hydrogen bonding, van der Waals forces, electrostatic interactions). Their core function lies in restricting the growth dimensions of perovskite materials, enhancing crystallinity, and improving material stability, while also conferring specific functionalities upon the perovskite.

#### 3.5.1. Polymer-Based Organic Templates

The long-chain molecular backbone of polymers can form physical constraint networks to precisely regulate perovskite crystal nucleation and growth. This refines grain size, reduces grain boundary defects, and induces preferred orientation. Concurrently, the coordination between polar groups and perovskite precursors optimizes film uniformity and density. The inherent flexibility of polymers also enhances the mechanical stability and durability of perovskite materials.

**Figure 6:**
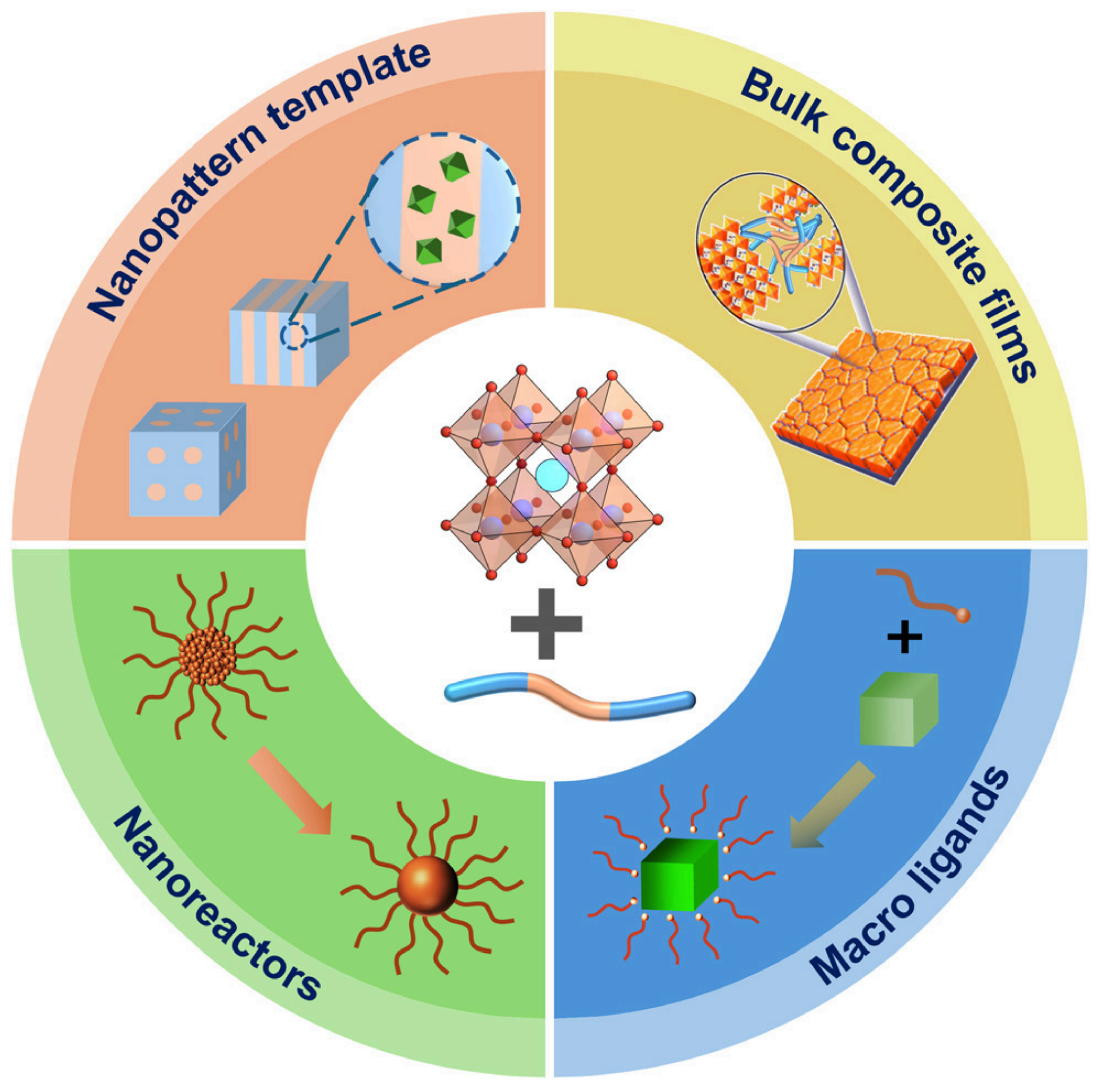
Overview of the different roles of BCP in perovskite–BCP hybrid structures [42].

As demonstrated by Liang et al.’s innovative use of organic templates for perovskite material synthesis, this research draws upon natural biomineralization mechanisms. The functional biopolymer carboxymethyl chitosan (CMC) has been introduced at the perovskite layer burial interface, thereby achieving Pb^2+^ enrichment through carboxymethyl group chelation and electrostatic interactions. Hydroxyl and amino groups form hydrogen bonds with I^−^ to inhibit ion migration, while O and N atoms undergo Lewis acid–base reactions with uncoordinated Pb^2+^ and Sn^4+^ ions to passivate interface defects. Likewise, the helical elastic structure of CMC mitigates residual stresses in polycrystalline films and suppresses deleterious interlayer reactions. This approach has been demonstrated to regulate uniform vertical nucleation and directional growth of perovskite crystals, thus optimizing film crystalline quality and energy-level matching. In addition, perovskite materials were synthesized using a non-reverse solvent technique. Employing an ITO/SnO_2_/CMC/perovskite/F-PEAI/Spiro-OMeTAD/Ag device architecture and optimizing CMC concentration (optimally at 1.0 mg/mL) resulted in the successful fabrication of high-performance perovskite films. Photovoltaic devices based on these films have been shown to achieve a high power conversion efficiency (PCE) of 25.12%, with a large-area device (1 cm^2^) reaching 24% PCE. They also demonstrated significantly enhanced stability under continuous illumination and humid conditions [40]. Shen et al. further employed block copolymerization as a templating strategy [41]. The chemical composition and chain structure designability of block copolymers (BCPs) were utilized to leverage their multifaceted roles within the composite system. These roles included utilization as nanostructuring templates, micelle/star-shaped crystallization templates, macromolecular ligands, encapsulation matrices, and bulk composite components, as shown in Figure 6. 

The inherent limitations of metal halide perovskites (MHPs)—namely poor stability and difficulty in nanostructuring—were addressed by leveraging specific interactions such as electrostatic forces, Lewis acid–base interactions and coordination bonds. This approach facilitated spatial control of perovskite nanostructures, regulation of crystallization, surface passivation, and environmental protection. In conclusion, five classes of perovskite–BCP composite structures were successfully constructed. It has been demonstrated that these significantly enhance the photothermal stability, moisture tolerance and dispersibility of perovskite materials while optimizing their optoelectronic properties—including light absorption, photoluminescence (PL) quantum yield and charge transport. Consequently, this has resulted in the development of flexible functional optical materials for solar cells (achieving a maximum photoconversion efficiency of 24.1%), LEDs, photodetectors, chemical/physical sensors, and bioimaging. This provides an efficient and viable solution for the extensive application of perovskites in optoelectronics [43]. In turn, Sun et al. innovatively employed amphiphilic block copolymers as organic templates. The amphiphilic block copolymer PS-b-P2VP self-assembles in toluene to form spherical micelles with a “PS shell–P2VP core” structure, thereby serving as soft templates. The multistage emulsion mechanism (comprising microemulsion and nanoemulsion) was employed to achieve uniform dispersion and complexation of precursors. The steric hindrance effect of the PS shell stabilizes PbBr_2_ microstructures within the microemulsion, whilst the pyridine groups of the P2VP core coordinate with Pb^2+^ ions, inducing PbBr_2_ dissociation and forming [PbBr_3_]^−^ complexes. Following a protracted period of stirring and centrifugal purification, these complexes are efficiently encapsulated by micelles. Due to CsBr’s suboptimal solubility in toluene, it is dissolved in methanol prior to being added dropwise to the system. The hydrophilicity of methanol facilitates the rapid diffusion of Cs^+^ ions to the P2VP core and [PbBr_3_]^−^, forming CsPbBr_3_ nanocrystals. It is imperative to exercise stringent control over the methanol dosage to circumvent any potential compromise to micelle stability. An excess of methanol has been observed to induce micelle fusion and the formation of [PbBr_4_]^2+^ complexes, thereby instigating Ostwald ripening of CsPbBr_3_ into CsPb_2_Br_5_ microcrystals that subsequently precipitate. In the perovskite materials that have been prepared, the incorporation of 10 μL of a 15 mg/mL CsBr methanol solution has been shown to yield CsPbBr_3_ nanocrystals that exhibit a cubic phase structure, the highest PLQY (84.46%), and the longest fluorescence lifetime (14.32 ns). It has been demonstrated that these crystals exhibit excellent optical stability, even after a storage period of four weeks at ambient temperature. It has been demonstrated that an excess of methanol, or a deficiency thereof, can result in distortions to the crystal structure, a deterioration of optical properties, and a reduction in long-term stability [42]. Finally, Wu et al. employed a polymer-based template strategy to synthesize high-quality perovskite materials. In their study, the researchers utilized triazine-based covalent organic frameworks (TPTP-COF, TPBT-COF, TPBTz-COF) as composite templates for perovskite crystal growth. The porous conjugated frameworks facilitate the formation of larger grains and higher-quality crystalline thin films by MAPbI_3_, whilst simultaneously passivating crystal defects, such as uncoordinated Pb^2+^ ions and vacancies, through chemical coordination. This process also optimizes charge separation and transport efficiency. Among these, TPBTz-COF exhibits stronger interface interactions with MAPbI_3_ due to its higher conjugation intensity and smaller particle size in the precursor solution, delivering the most favorable templating effect and defect passivation. Key achievements include: In the present study, the photovoltaic conversion efficiency of TPBTz-COF-modified trans-perovskite solar cells was found to be 20.04% (open-circuit voltage 1.04 V, short-circuit current density 24.26 mA·cm^−2^, fill factor 79.40%) [43]. The cells demonstrated excellent retention of efficiency, with 80% of the initial efficiency being maintained after 400 h of storage at 30 °C and 60% relative humidity. Similarly, the crystal diffraction intensity, photoluminescence intensity, carrier lifetime, and charge mobility of the MAPbI_3_ film were significantly enhanced in comparison with pure MAPbI_3_ films, thus demonstrating the critical value of COF templating in improving the photovoltaic performance and stability of perovskite materials.

#### 3.5.2. Alkylammonium-Based Organic Templates

Alkylammonium compounds represent highly practical organic templates, offering advantages in molecular structural designability and exceptional compatibility with inorganic frameworks. Precise control over alkyl chain length enables dimensional regulation of perovskite materials; shorter chains support continuous 3D inorganic frameworks, conferring benefits such as high carrier mobility and superior optoelectronic properties.

**Figure 7:**
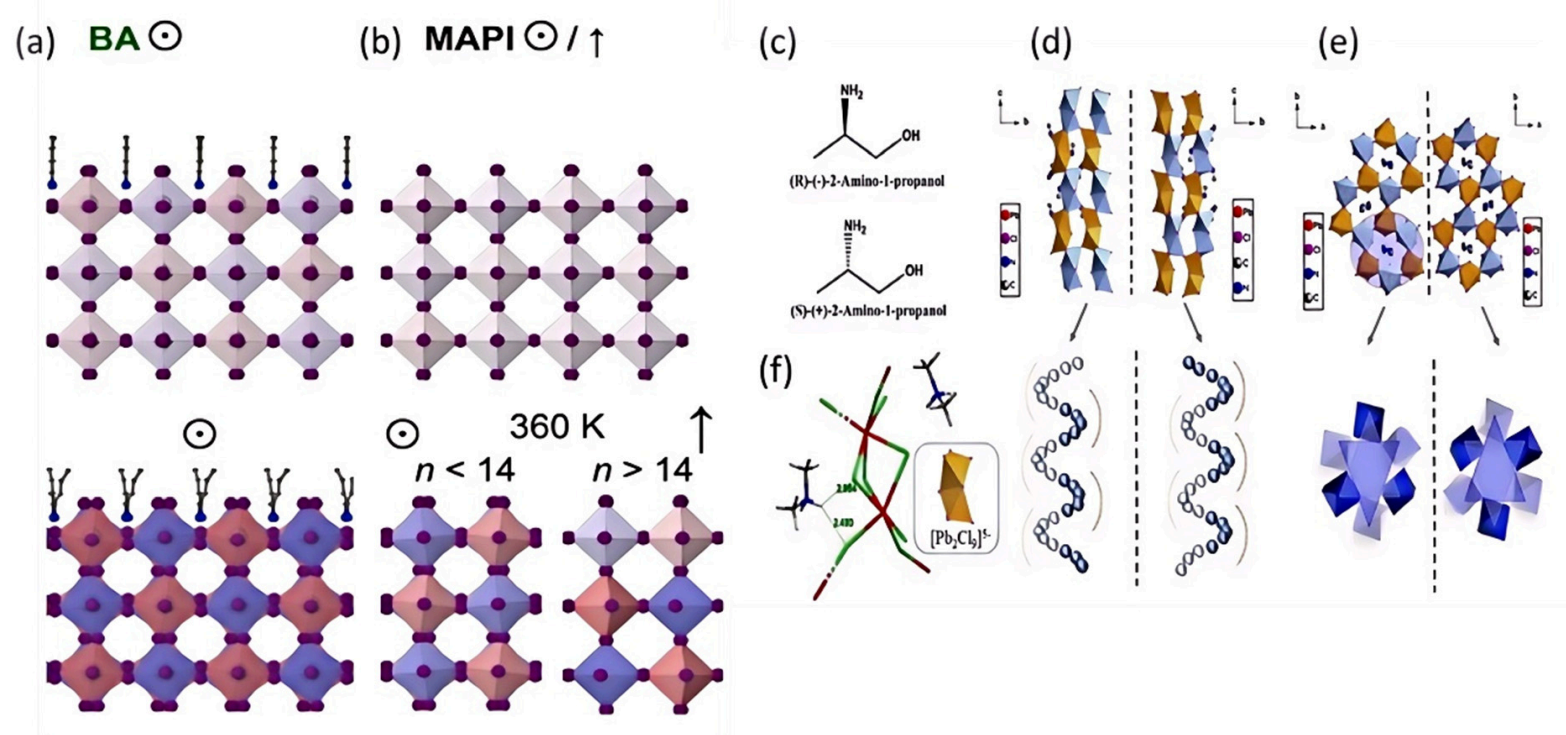
(**a**) BA-based 2D HPs, as well as (**b**) MAPbI_3_ surfaces. Triangles and circles indicate the transition temperatures for the surface and interior layers, respectively [44]. (**c**) The chiral inducers studied of 1-R/S. (**d**) Crystal structure of 1-R/S along the b-axis, the [Pb_2_Cl_9_]^5−^ dimer, and a schematic representation of their localized structures. (**e**) Crystal structure of 1-R/S along the c-axis and a schematic representation of their localized structures. (**f**) Localized detail of DMA^+^ with [Pb_2_Cl_9_]^5−^ [45] Copyright 2024, ACS.

As demonstrated by Fransson et al. in synthesizing perovskite materials using organic ammonium templates, the chemical properties (volume, structure) of the template molecules (including phenethylammonium PEA, phenylmethylammonium PMA, butylammonium BA, methylammonium MA, etc.) synergistically modulate the perovskite layer count (dimension), enabling precise templating of two-dimensional halide perovskite phase transitions and octahedral tilting behavior (Figure 7a,b) [44]. Large-volume organic ammonium templates (PEA, PMA) significantly elevate the phase transition temperature of the surface layer in direct contact with the template compared to the bulk MAPbI_3_, while small-volume organic ammonium templates (MA) lower the surface layer transition temperature below that of the bulk phase. Medium-volume organic ammonium templates BA demonstrate no discernible distinction between surface and bulk transitions, with inner-layer transitions approaching bulk characteristics as the perovskite layer count increases. Similarly, the octahedral tilting axis (in-plane/out-of-plane) and tilt angle exhibit systematic variations depending on the organic ammonium template type. It is evident that the core achievements of this research are founded on the following mechanisms: firstly, the establishment that organic ammonium molecules function as pivotal templates, thereby regulating the structure and properties of two-dimensional perovskites; secondly, the revelation of the dual phase transition mechanism that is mediated by these templates in both the surface and bulk layers of perovskites; thirdly, the establishment of the “organic ammonium template chemistry–dimension–octahedral tilt—electronic structure” correlation chain; fourthly, the elucidation of the spatial distribution patterns of the valence band maximum (VBM) and conduction band minimum (CBM) under template-controlled structural dynamics; and finally, the proposal of a universal design strategy to tailor the inorganic dynamics and optoelectronic properties of two-dimensional perovskites across a broad temperature range by selecting specific organic ammonium templates and controlling perovskite layer thickness. This provides crucial theoretical support for the development of highly efficient and stable perovskite optoelectronic devices (such as solar cells, lasers, and light-emitting diodes). Likewise, the work by Yang et al. demonstrates remarkable innovation, as shown in Figure 7c–f [45]. The research employs organic amines as templates (non-typical organic ammonium compounds), utilizing chiral organic amines or alcohols as templates. In the presence of an acidic environment, these substances undergo a reaction with PbCl_2_ and a N, N-dimethylformamide (DMF) system. This approach generates chiral precursors through interactions between the templating agent and PbCl_2_, followed by self-assembly of DMA^+^ cations with these precursors. The construction of a three-dimensional perovskite structure containing helical Pb_2_Cl_9_^5−^ chains is achieved through the utilization of N-H—Cl hydrogen bonds and Coulombic interactions. The template agent has not yet entered the lattice, yet it is able to determine the R/S configuration of the product, thereby achieving precise transfer and control of chirality. Their achievements include the successful development of a universal, controllable method for synthesizing chiral 3D perovskites. The R/S-[DMA]PbCl_3_ material exhibits a pure-phase structure, thermal stability below 242 °C and mirror-symmetric circular dichroism signals across the 210–390 nm wavelength range, demonstrating strong absorption characteristics in the ultraviolet region. The investigation revealed that circularly polarized light detectors constructed from this material exhibited a photocurrent anisotropy factor of 0.296. This result matched the best performance of existing 3D perovskite devices of the same type. Furthermore, the study demonstrated the stability and reproducibility of the detection response, thus establishing a novel method for the synthesis of chiral 3D perovskites and their utilization in the detection of ultraviolet circularly polarized light [45].

#### 3.5.3. Organic Templates Based on Zwitterionic Ions

Amphoteric ions represent another highly promising class of organic templates, offering synergistic cation-anion interactions within the molecule and multi-site defect passivation capabilities. Molecules bearing both positive and negative charge groups (e.g., sulfonate/ammonium, carboxyl groups/imidazole groups, etc.) can precisely passivate positive and negative charge defects such as Pb^2+^ and halide vacancies in the perovskite bulk and at interfaces through multiple interactions including ionic bonds, coordinate bonds, and hydrogen bonds. This suppresses non-radiative recombination and enhances carrier mobility. Polymeric materials also represent highly competitive organic templates, endowing perovskite materials with high functionalization and entrepreneurial capabilities.

**Figure 8:**
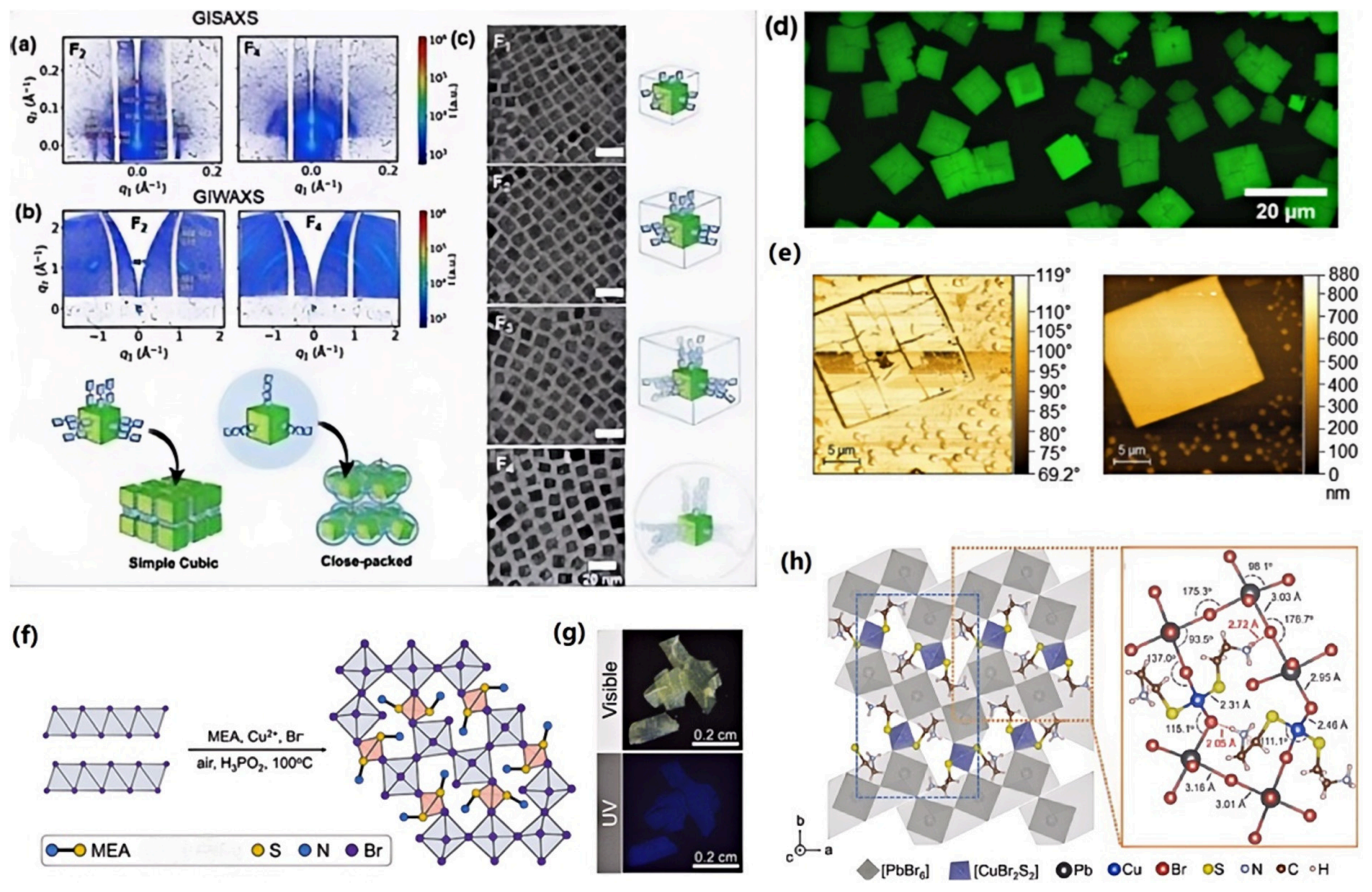
(**a**) GISAXS measurements of F^n-^SB@CsPbBr_3_ NCs n = 1–4 prepared via ligand exchange on OA@CsPbBr_3_ NCs, cast as a film on the silicon substrate, which show a gradual shift in the distance between packing planes in the in-plane scattering direction as well as a transition from simple cubic to close-packed arrangement of the NCs in the film. (**b**) Azimuthal broadening for the F^4-^SB@CsPbBr_3_ NC film suggests that the longer ligand disrupts any orientational preference of the NC with respect to the underlying substrate. (**c**) TEM images of these same NCs showing inter-NC distance to increase with ligand length (**d**) Confocal fluorescence microscopy images of superstructures formed from F^2-^SB@CsPbBr_3_ NCs. Polarized optical light microscopy image. (**e**) giving approximate dimensions of 16 μm × 19 μm × 0.5 μm for the assembled structure [47] Copyright 2024, ACS. (**f**) Schematic depiction of the synthetic pathway. (**g**) Stereo fluorescence microscope images of the single crystals under visible light and 365 nm ultraviolet (UV) light irradiation. (**h**) Single-crystal structure of (MEA)_4_Cu_2_Pb_3_Br_12_. The unit cell is highlighted by the blue rectangle. Zoom-in of the orange rectangle with key geometrical parameters [48] Copyright 2024, ACS.

As with Cueto et al., the present study employs a template-like approach to design and synthesize chain-terminated fullerene conjugated ligands (F_n_-SB, n = 1–4) bearing sulfonic acid betaine zwitterions via iterative Suzuki coupling and chain-terminated functionalization reactions [46]. The purpose of this study is to ascertain the means by which stable binding is achieved, through strong chelation between the zwitterionic headgroup and perovskite surface A-site and X-site vacancies. The conjugated backbone ensures charge transport capability, while the n-octyl side chain adapts to low-polarity solvent systems. The precise control of the ligand chain length enables the regulation of the inter-nanocrystal spacing and stacking geometry of perovskite nanocrystals (NCs). Achievements in perovskite material synthesis include the successful acquisition of F^n-^SB@CsPbBr_3_ composite nanocrystals via direct synthesis or ligand exchange methods, as shown in Figure 8a–c. The material displays a high fluorescence quantum yield (PLQY > 90%), demonstrating remarkable stability under thermal and polar solvent conditions. Notably, F4-SB modified samples exhibit a notable retention of high PL after undergoing 120 °C annealing or methanol immersion, underscoring their resilience to external stress. This facilitates the systematic control of nanocrystal spacing ranging from 1.0 nm to 4.1 nm and transitions in stacking patterns from simple cubic to hexagonal close-packed structures. Furthermore, these materials have the capacity to self-assemble into micrometer-scale ordered superstructures. It has been demonstrated that there is efficient electronic communication and excited-state energy transfer between ligands and nanocrystalline cores. This provides novel hybrid materials for optoelectronic applications, combining stability, conductivity and structural tunability. In a similar manner, Zhao et al. employed an innovative approach mediated by zwitterionic ligands, utilizing mercaptoethylamine (MEA) as a bifunctional ligand [47]. In acidic conditions, the -NH^3+^ group of its zwitterionic form (-S-[CH_2_CHNH^3+^]) interacts with the [PbBr_6_]^4−^ octahedron to form hydrogen bonds, while the S atom coordinates with Cu(I). This process successfully integrates the [CuS_2_Br_2_] tetrahedral cluster into the perovskite structure [PbBr_6_]^4−^ octahedra via hydrogen bonding, while the S atom coordinates with Cu(I). This integration process effectively incorporates [CuS_2_Br_2_] tetrahedral clusters into the lead halide perovskite three-dimensional (3D) framework, thereby yielding the novel bimetallic perovskite material (MEA)_4_Cu_2_Pb_3_Br_12_, as shown in Figure 8d,e. This approach effectively addresses the challenge of phase separation or component exchange when large-volume building units co-crystallize with perovskite frameworks. The material under scrutiny in this study exhibits a compact, stable crystal structure (thermal stability up to 408 K in nitrogen atmosphere), displays macroscopic diamagnetism, and maintains stability across the 300–500 K temperature range. The formation of acceptor levels occurring at a level above the valence band top has been demonstrated to promote photogenerated carrier separation, thus resulting in dual strong absorption characteristics in the visible light spectrum (380–550 nm) and the near-infrared spectrum (1500–2700 nm). In turn, the material displays narrow-peak photoluminescence at 406 nm (15 nm full-width at half-maximum) and a fluorescence lifetime of 1.30 ns, thus providing a novel approach for the design of perovskite derivatives with programmable optoelectronic properties, as shown in Figure 8f–h [48].

### 3.6. Composite Templated Perovskite Materials

Hybrid templating combines the advantages of two or more individual templates. Through structural and functional synergy, it addresses challenges in perovskite material synthesis, including morphology control, crystallization regulation, stability enhancement and interface adaptation. This represents a highly valuable synthetic strategy for perovskite materials.

Using a composite template strategy, Li et al. utilized the benefits of polymer templates in conjunction with graphite’s advantages to create a graphene-poly(methyl methacrylate) (PMMA) composite structure. This composite structure functioned as a primary template, exhibiting synergistic reinforcement effects. Graphene, characterized by its exceptional mechanical strength (tensile strength of 130 GPa, Young’s modulus of approximately 1 TPa), provides rigid constraints to the perovskite lattice [14]. PMMA, in this context, serves as a coupling template. The presence of acrylic ester groups within the structure of the material has been demonstrated to facilitate the formation of strong bonds with lead cations on the perovskite surface. This, in turn, has been shown to enhance the interfacial adhesion between graphene and perovskite, whilst simultaneously suppressing light-induced lattice dynamic expansion. This composite template has been demonstrated to passivate defects, impede ion migration and provide physical protection for the perovskite film. Significant achievements have been attained in the field of perovskite material and device preparation, including the enhancement of the Young’s modulus and hardness of perovskite films, and the attainment of a power conversion efficiency (PCE) of 24.6% for solar cells based on this material, with some systems demonstrating a higher efficiency of up to 25.4%. In addition, the experiment was conducted at a temperature of less than 90 °C under AM 1.5 G illumination for a period of 3670 h. The device demonstrated exceptional stability under a range of stringent conditions, including unencapsulated ambient environments and low vacuum. This overcomes the challenges of lattice deformation and device degradation inherent to traditional perovskite materials due to their soft-ion characteristics, laying a crucial foundation for the industrial application of perovskite solar cells.

MOFs have emerged as a highly competitive functional material and a prominent research focus, demonstrating an expanding role in the domain of perovskite materials. As demonstrated by Dong et al., the use of MOF-808 as the core template resulted in the uniform in-situ growth of COF on its surface via covalent bonding, thereby forming core-shell structured MOF@COF nanoparticles. The MOF-808 template not only effectively suppressed face-to-face stacking and agglomeration of the COF but also provided a stable growth platform for the COF through its porous structure and active sites. Similarly, the substance exhibited a high degree of affinity for heavy metal ions, thereby facilitating the capture of lead ions. The formation of heterogeneous pre-nucleation centers is a consequence of strong chemical interactions between surface C-N, -COO^−^ and C=O functional groups. This process led to the optimization of the crystalline quality of large-grain perovskite films, the elimination of deep-level defects and the simultaneous “capture” of leaking lead ions through in situ chemical fixation and adsorption. 

**Figure 9:**
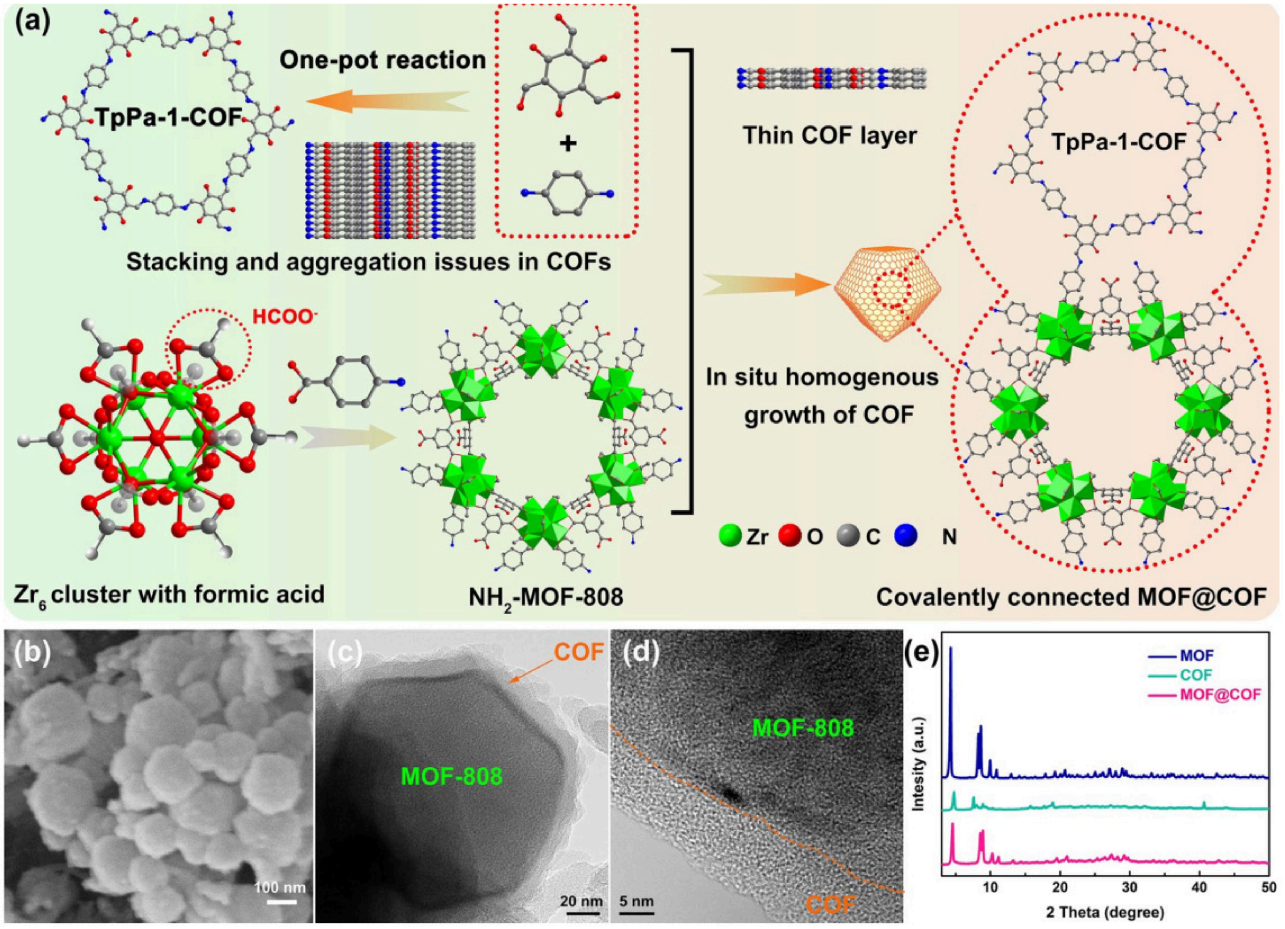
(**a**) Preparation process of the MOF@COF. Scanning electron microscopy (SEM) images of (**b**) MOF@COF. (**c**) Transmission electron microscopy (TEM) and (**d**) high-resolution (HR)-TEM images of MOF@COF. (**e**) XRD patterns [48].

The study concluded with the attainment of noteworthy outcomes: Perovskite solar cells constructed with this material exhibited a photovoltaic conversion efficiency of 23.61%, which is commendable, along with an open-circuit voltage of 1.20 V, indicative of their effectiveness. The devices that were not encased retained approximately 90% of their initial efficiency after undergoing 2000 h of operation under conditions of 30–50% relative humidity (RH) and 25–30 °C, with lead leakage levels measuring less than 5 parts per million (ppm). These findings align with the environmental assessment standards, thereby confirming the safety and sustainability of the material. This finding underscores the pivotal role of MOF templates in constructing multifunctional composite systems that simultaneously address perovskite device efficiency, stability and environmental safety concerns, as shown in Figure 9.

### 3.7. Template Removal Methods and Technical Challenges

Template removal is an essential post-treatment procedure in template-assisted perovskite fabrication, which directly determines final film morphology, residual impurity content, interfacial quality, and overall device performance. Rational removal strategies should fully preserve the optimized perovskite crystal structure while eliminating template residues without introducing additional defects or structural collapse. Current mainstream template removal approaches can be classified into thermal calcination, solvent etching, chemical dissolution, and physical stripping, each with distinct applicable scenarios and limitations.

Thermal calcination is the most widely adopted method for inorganic and soft organic templates. Organic templates such as surfactants, polymer micelles, and small-molecule alkylammonium compounds can be completely decomposed and removed under controlled high-temperature annealing (300–500 °C). Carbon-based templates can also be eliminated via high-temperature oxidation. Nevertheless, excessive calcination temperature easily induces perovskite phase transition, lattice distortion, and thermal decomposition, especially for hybrid organic–inorganic and lead-free perovskites with poor thermal stability. Precise temperature and atmosphere control are therefore required to balance template elimination and crystal integrity.

Solvent etching represents a milder low-temperature route, particularly suitable for thermally sensitive perovskite systems. Polar and nonpolar mixed solvents can selectively dissolve organic polymer templates and partial oxide sacrificial layers without damaging the perovskite framework. This solution-based process features simple operation and low thermal impact, yet it risks inducing ion dissolution, grain corrosion, and residual organic impurities if solvent compatibility is not well matched. Optimized solvent ratio and immersion time are critical to avoid structural degradation.

Chemical dissolution is primarily applied to inorganic silica-based templates such as SBA-15 and MCM-41. Weak alkali or dilute HF solutions can selectively etch silica frameworks while maintaining perovskite integrity. However, chemical etching inevitably introduces residual ions and interfacial defects, and strict post-washing treatment is required to eliminate etching byproducts, which increases process complexity and limits large-area batch fabrication.

Physical stripping and sacrificial layer peeling are commonly used for ordered hard templates such as AAO and 2D layered substrates. By introducing water-soluble or easily peeled sacrificial interlayers, the template can be mechanically separated from the perovskite film after crystallization. This method enables residue-free removal and maintains intact grain arrangement, whereas it is only applicable to specific ordered nanostructures and difficult to popularize for conventional polycrystalline films.

From the perspective of industrialization, existing template removal techniques still face prominent bottlenecks: high-temperature calcination is incompatible with flexible devices and thermolabile perovskites; solvent and chemical etching introduce impurities and complicate the process; physical stripping lacks universal adaptability. Developing mild, residue-free, low-cost, and large-area compatible template removal technology remains an important prerequisite for the practical application of template-assisted perovskite photovoltaics. Future research should focus on designing easily removable template structures and developing green one-step removal strategies compatible with roll-to-roll manufacturing.

Moreover, we have supplemented a dedicated comparative table to clearly summarize the advantages, limitations, applicable scenarios and industrialization potential of each template type, as shown in Table 1. This comparative analysis enables readers to intuitively grasp the characteristics and applicable scope of different template methods, and significantly enhances the logicality and academic depth of this review.

From a critical perspective, existing research on template-assisted perovskite synthesis mostly focuses on independent experimental results of individual materials, while horizontal comparison among different template categories is insufficient. Inorganic templates excel in lattice matching and spatial confinement but lack interfacial functional modulation capability; organic templates show outstanding defect passivation and molecular regulation effects, but suffer from poor thermal stability and weak structural constraint ability. Such inherent trade-offs are rarely systematically discussed in current literature.

In addition, some inconsistent conclusions exist in reported studies. For example, the optimal pore size of inorganic templates and the optimal molecular chain length of organic templates show obvious differences in different literatures, which may be attributed to different precursor systems, preparation conditions and characterization methods. At present, there is still a lack of unified design criteria and consensus mechanism interpretation.

In terms of practical technical bottlenecks, most reported high-performance templates rely on expensive raw materials, complex synthesis procedures and non-recyclable characteristics, which are difficult to adapt to blade coating and roll-to-roll industrial manufacturing. Moreover, most studies focus on small-area laboratory devices, while the stability and reproducibility of the template strategy in large-area modules remain insufficiently explored. Composite templates are regarded as the most promising direction to balance performance and stability, but their component compatibility and scalable preparation technology still need further breakthroughs in future research.

### 3.8. Template Method of Lead-Free Perovskites

Given the environmental toxicity of lead-based perovskites, the development of tin (Sn)-, germanium (Ge)-, bismuth (Bi)- and antimony (Sb)-based lead-free perovskites has become an inevitable trend for future commercialization. Nevertheless, lead-free systems still face severe intrinsic limitations, including facile oxidation of Sn^2+^, poor phase stability of Ge-based perovskites, wide bandgap and high defect density in Bi/Sb-based derivatives, as well as uncontrollable nucleation and grain growth during conventional solution processing. In this regard, template-assisted strategies offer a promising route to address these bottlenecks by regulating crystal orientation, suppressing phase transition, passivating interfacial defects, and improving ambient stability [49,50,51,52].

Tin-based perovskites (e.g., CsSnX_3_, FASnI_3_) are the most widely investigated lead-free candidates owing to their suitable bandgap and high carrier mobility. Inorganic templates such as SiO_2_, AAO, and 2D perovskite frameworks can confine crystal growth, inhibit the oxidation of Sn^2+^, and reduce non-radiative recombination. Organic and polymer templates further homogenize precursor distribution, passivate grain boundaries via hydrogen bonding and coordination interactions, and effectively improve the reproducibility of large-area films.

Germanium-based perovskites possess low toxicity and ideal optoelectronic properties but suffer from poor crystallinity and structural instability. Template-induced lattice matching and heterogeneous nucleation can optimize grain arrangement, relieve lattice distortion, and enhance the thermal and moisture resistance of Ge-containing perovskite absorbers.

Bismuth- and antimony-based perovskites (such as Cs_2_AgBiBr_6_ and Cs_3_Sb_2_I_9_) exhibit excellent ambient stability and low toxicity, yet their intrinsically wide bandgap and high trap density restrict photovoltaic performance. Composite templates constructed from MOFs, COFs, and organic ligands have been employed to modulate their nucleation kinetics, optimize film morphology, and reduce defect states, providing an effective pathway toward high-efficiency lead-free photovoltaic devices.

Overall, the template method has demonstrated unique advantages in solving the core challenges of lead-free perovskites, including vulnerable chemical stability, inferior crystalline quality, and high defect density. Future efforts should focus on developing low-cost, recyclable template materials, establishing universal template design principles for Sn/Ge/Bi/Sb systems and promoting the scalable fabrication of stable and efficient lead-free perovskite solar cells.

### 3.9. Compatibility of Template Strategies with Industrial Scalable Coating Methods

The commercialization of perovskite photovoltaic technology inevitably requires the transition from laboratory spin-coating to large-area industrial manufacturing techniques, among which blade coating, slot-die coating, and roll-to-roll (R2R) processing are the most promising mainstream fabrication routes. Template-assisted synthesis, as an effective strategy to regulate perovskite crystallization, must maintain good compatibility with these scalable processes to realize practical industrial deployment.

In terms of blade coating, solution-processed organic and polymer templates exhibit excellent adaptability. Long-chain surfactants, functional polymer additives, and small-molecule organic templates can be uniformly dispersed in perovskite precursor inks, effectively regulating ink rheology, optimizing wet-spreading behavior, and suppressing rapid random nucleation during blade shearing and solvent evaporation. Such template effects alleviate common large-area fabrication issues including film inhomogeneity, pinholes, and grain inhomogeneity. In contrast, rigid inorganic templates with bulky solid structures are difficult to disperse uniformly in precursor solutions, and their rigid frameworks easily cause coating streaks and surface roughness, showing limited compatibility with blade-coating production lines.

For slot-die coating and roll-to-roll continuous manufacturing, low-viscosity ink compatibility and rapid in-situ crystallization requirements become more stringent. Molecular-level organic templates and ultra-thin 2D perovskite template layers are highly suitable for R2R processes. They can be dissolved in low-polarity green solvents, match the high-speed web coating condition, and induce oriented crystal growth during low-temperature inline annealing. Composite templates constructed by organic functional layers coupled with inorganic nano-scaffolds also show great application potential; they simultaneously regulate ink fluidity and crystal orientation without affecting continuous roll-to-roll deposition efficiency. However, traditional high-temperature calcined inorganic templates and thick ordered frameworks (such as large-pore AAO) are incompatible with flexible substrate roll-to-roll processes and cannot adapt to low-temperature continuous production requirements.

From the perspective of industrial scalability and cost-effectiveness, organic molecular templates and thin-layer 2D perovskite templates are currently the most feasible options for large-area manufacturing. They feature low raw material cost, simple dosing operation, good solvent compatibility, and no need for complex post-treatment. Composite templates are regarded as the key future development direction because they can balance crystallization quality, device stability and process compatibility. In contrast, most rigid inorganic templates still face obvious limitations in ink dispersion, continuous coating adaptability and mass production cost. Future template design should further focus on low-viscosity ink compatibility, low-temperature crystallization adaptability, and green solvent solubility, so as to fully match the technical requirements of blade coating, slot-die and roll-to-roll industrial production lines.

### 3.10. Template Strategies for Perovskite–Silicon Tandem Solar Cells

Current review discussion of templated perovskite photovoltaic devices mainly focuses on single-junction perovskite and all-perovskite tandem architectures, while overlooking perovskite–silicon tandem solar cells—the most technically mature route with certified efficiency exceeding 34.85% and the closest to industrial mass production. Silicon bottom subcells impose unique optical confinement, interface energy-level matching, and low-temperature crystallization constraints on the top perovskite absorber layer, which puts forward distinct requirements for template design compared with pure perovskite films. This subsection systematically supplements template engineering strategies tailored for perovskite–silicon tandem devices, covering self-assembled monolayer (SAM) molecular templates, black silicon nano-scaffold hard templates, and additive-mediated chemical induction templating, alongside their dual-phase nucleation regulation mechanisms.

#### 3.10.1. Self-Assembled Monolayers (SAMs) as Molecular Templates for Silicon/Perovskite Interfaces

Self-assembled monolayers act as ultrathin molecular templates at the silicon/perovskite heterointerface, simultaneously functioning as heterogeneous nucleation sites and interfacial dipole regulators. SAM molecules with silane anchoring groups covalently bond to the oxidized silicon surface, while terminal ammonium, carboxylate or thiol groups interact with perovskite precursor ions via hydrogen bonding and Lewis acid–base coordination, realizing molecular-scale confinement for perovskite nucleation. As reported in ref. [53], amino-silane SAM templates pre-modified on textured silicon wafers homogenize local precursor supersaturation, suppress random island-like perovskite nucleation, and induce uniform lateral grain growth across micrometer-scale silicon pyramids. The monolayer template eliminates high-density dangling bond defects at the inorganic heterojunction, boosts open-circuit voltage by over 70 mV and maintains 92% initial efficiency for encapsulated tandem modules under 85 °C/85 RH aging. Another study DOI:10.1039/D5EL00089K further develops zwitterionic SAM molecular templates: dual charged sulfonate-ammonium backbones passivate both uncoordinated Pb^2+^ and halide vacancies at the perovskite bottom surface, while the ultra-thin template layer avoids parasitic light absorption, breaking the traditional trade-off between interface defect passivation and short-circuit current density. Different from bulk organic polymer templates, SAM molecular templates possess sub-2 nm thickness without residual organic barrier layers, perfectly matching the low light-loss demand of tandem bottom silicon subcells.

#### 3.10.2. Black Silicon Nanoscale Scaffold Hard Templates

Black silicon (BSi) fabricated via reactive ion etching or metal-assisted chemical etching serves as a three-dimensional inorganic nano-scaffold template for perovskite–silicon tandems, combining anti-reflection optical confinement and spatial crystallization regulation functions. The dense, disordered silicon nanopore array acts as a rigid confinement space to restrict perovskite grain overgrowth, relieve lattice mismatch strain between the textured silicon substrate and perovskite thin film, and extend optical absorption path length of the top cell. Multiple works have verified the advantages of black silicon template engineering in tandem devices [54,55,56]. The nanopore size of black silicon templates can be precisely tuned from 20–200 nm; pores below 50 nm provide strong spatial confinement to refine perovskite grain size and reduce grain boundary recombination, while 100–200 nm pore channels realize full precursor infiltration for thick perovskite light-absorbing layers. The rigid silicon scaffold template exhibits excellent high-temperature resistance, compatible with post-annealing procedures of wide-bandgap perovskites (1.65–1.78 eV) used for tandem top cells. Meanwhile, the black silicon template eliminates additional anti-reflection coating layers, simplifying the tandem device stack structure. The main limitation lies in uneven precursor infiltration in deep nanopores during large-area blade coating, which requires composite modification with low-molecular-weight organic co-templates to optimize ink wettability.

#### 3.10.3. Additive-Mediated Chemical Induction Templating for Two-Phase Perovskite Crystallization on Silicon Textures

For solution-processed perovskite films deposited atop textured silicon surfaces, severe precursor concentration gradients easily trigger heterogeneous two-phase crystallization, generating mixed low-quality intermediate phases and high trap density. Additive chemical induction templating introduces trace template molecules into perovskite inks, constructing transient heterogeneous nucleation centers to regulate sequential two-phase transformation, and retains residual template effects to passivate final polycrystalline films. Two representative template additive systems are summarized here [57]: (1) Tin oxide nanocluster in-situ seed templates: trace SnO_2_ colloidal additives form lattice-matched nucleation seeds on silicon pyramids, guiding the transition from PbI_2_-DMSO intermediate phase to pure perovskite phase, suppressing residual lead iodide impurity phases that cause subcell current mismatch in tandems; (2) Alkylammonium halide small-molecule transient templates: short-chain MA/FA ammonium ions act as temporary confinement templates during solvent evaporation, homogenize vertical crystal growth on uneven silicon textures, and partially volatilize during mild annealing with residual trace components passivating grain boundaries. Compared with template-free two-step deposition on silicon, additive chemical templating reduces non-radiative recombination loss by over 40%, and the fabricated perovskite–silicon tandem devices achieve steady-state efficiency above 33%.

#### 3.10.4. Compatibility and Development Outlook of Templates for Perovskite–Silicon Tandems

Inorganic hard templates (black silicon, oxide seeds) excel in optical management and thermal stability but face poor ink infiltration uniformity for textured silicon substrates; SAM molecular templates achieve atomically smooth heterointerfaces with negligible optical loss yet lack three-dimensional confinement capacity; organic additive transient templates show outstanding large-area coating compatibility but require precise dosage control to avoid phase segregation. Composite template systems integrating SAM molecular underlayers, silicon nano-scaffolds, and trace additive regulators are regarded as the optimal solution for future high-efficiency tandem modules.

Unlike all-perovskite tandem devices, perovskite–silicon stacks demand template materials free of heavy metal contamination and high parasitic absorption. Future template design for silicon-based tandems should prioritize ultra-thin, low-residue, silicon-texture-adaptable molecular and nano-scaffold templates, and develop roll-to-roll compatible in-situ templating deposition technology to push the commercialization of perovskite–silicon tandem photovoltaic products.

## 4. Conclusions

This review systematically summarizes the research progress of inorganic, organic, and composite template strategies in regulating the crystallization behavior and microstructure evolution of perovskite nanomaterials, as well as their photovoltaic performance modulation mechanisms. The intrinsic structure–performance relationship induced by different template types is clearly clarified. Inorganic templates optimize grain orientation, alleviate lattice strain and reduce intrinsic defects via spatial confinement and lattice epitaxial effects, effectively improving the thermal and structural stability of perovskite devices. Organic templates regulate perovskite nucleation and growth through hydrogen bonding, electrostatic interaction and Lewis acid–base coordination, which enables grain refinement, interfacial defect passivation and ion migration suppression, and further enhances the mechanical flexibility and environmental robustness of perovskite films. Composite templates integrate the superiorities of inorganic rigid frameworks and organic functional components, achieving synergistic optimization in crystalline quality, optoelectronic properties and lead leakage suppression, thereby providing a feasible route to balance photovoltaic efficiency, operational stability and environmental safety. We note that most current template studies are concentrated on MAPbI_3_ and FAPbI_3_ lead-based perovskite systems, which exhibit typical crystallization behavior and defect evolution characteristics. However, such findings cannot be directly generalized to tin-based, all-inorganic CsPbX_3_ and Bi/Sb-based lead-free perovskites, due to their distinct ionic radii, electronic configurations, crystallization kinetics, and intrinsic stability limitations. Therefore, the regulatory mechanism and applicable scope of each template type must be discussed separately for different perovskite categories, with sufficient literature support for non-lead systems. Recent advances in template engineering for Sn-based perovskites, all-inorganic halide perovskites, and vacancy-ordered Bi/Sb perovskites are further supplemented and analyzed in this review, enabling a more objective and classified discussion rather than simple overgeneralization.

Although the template method has achieved remarkable advances in perovskite photovoltaics, several critical bottlenecks still hinder its further development and industrialization. At present, most reported templates suffer from high raw material cost, complicated preparation procedures and poor recyclability, which are difficult to adapt to large-scale manufacturing requirements. In addition, the underlying regulation mechanisms of numerous template systems remain unclear, and unified and universal template design principles are still lacking, which limits the rational selection and targeted optimization of template materials. Most existing studies concentrate on lead-containing perovskite systems, while template exploration for tin-, germanium-, bismuth- and antimony-based lead-free perovskites is still inadequate. Moreover, the compatibility of current template technologies with low-cost large-area fabrication routes such as blade coating and roll-to-roll processing needs to be further improved.

Future research should prioritize the following directions. It is urgent to develop low-cost, eco-friendly and recyclable inorganic–organic composite templates, and establish universal design criteria matching perovskite lattice structure and growth characteristics. Fundamental investigations should be strengthened to reveal the microscopic regulation mechanisms of various templates at the atomic and molecular levels, so as to construct targeted modulation strategies for defect suppression and carrier dynamics optimization. More attention should be paid to template engineering of lead-free perovskites, focusing on restraining ion oxidation and optimizing phase stability to accelerate the practical deployment of non-toxic photovoltaic devices. Furthermore, it is necessary to promote the integration of template strategies with two mainstream tandem architectures: all-perovskite stacked devices and record-efficiency perovskite–silicon tandem cells. For silicon-based tandem photovoltaics, targeted template engineering including self-assembled monolayer (SAM) interfacial molecular templates, black silicon three-dimensional nano-scaffold hard templates, and additive-mediated transient chemical induction templates should be systematically developed to resolve heterojunction lattice mismatch, two-phase crystallization inhomogeneity and optical loss bottlenecks on textured silicon substrates. Optimizing the compatibility of the template method with industrial large-area fabrication processes such as blade coating and roll-to-roll processing will lay a solid foundation for the future commercialization of high-efficiency and stable single-junction and tandem perovskite solar cells.

## Figures and Tables

**Table 1:** Comparison of advantages, limitations and application prospects of inorganic, organic and composite templates for perovskite crystallization regulation.

Template Type	Advantages	Limitations	Main Application Scenarios	Industrialization Potential
Inorganic Templates	High rigidity, good lattice matching, excellent thermal/chemical stability, obvious spatial confinement effect, easy to realize oriented crystal growth, high carrier lifetime	Relatively single function, poor interface compatibility with organic perovskite layers, partial templates with high preparation cost, difficult to achieve precise regulation at molecular level	High-stability devices, 2D/3D perovskite heterostructures, high-temperature resistant photovoltaic devices	Medium; limited by cost and poor interface tailorability
Organic/Polymer Templates	Rich functional groups, flexible molecular design, excellent defect passivation ability, good film flexibility, low solution-processing requirement	Weak rigid confinement, poor high-temperature resistance, prone to introducing organic residual impurities, inevitable ion migration under long-term operation	Flexible perovskite devices, low-temperature solution processing, defect modulation through engineering	Medium–high; suitable for roll-to-roll large-area fabrication
Composite Templates	Synergistic advantages of inorganic rigidity + organic functionalization, simultaneous optimization of crystallinity, defects and interface energy-level alignment, strong comprehensive regulation ability	Complex preparation process, difficult to precisely control component ratios, relatively few systematic studies	Tandem solar cells, lead-free perovskite devices, high-efficiency and stable photovoltaic modules	High; most promising for balancing performance and scalability

## Data Availability

No new data were created or analyzed in this study. Data sharing is not applicable to this article.
